# Determination of Kinematic Viscosity of Mg(ClO_4_)_2_ and KOH Brines Saturated with CO_2_ at Sub-Zero Temperatures

**DOI:** 10.3390/molecules28155641

**Published:** 2023-07-25

**Authors:** Elizabeth Sargeant, Paramaconi Rodriguez

**Affiliations:** 1 School of Chemistry, University of Birmingham, Birmingham B15 2TT, UK; 2 Centre for Cooperative Research on Alternative Energies (CICenergiGUNE), Basque Research and Technology Alliance (BRTA), Alava Technology Park, 01510 Vitoria-Gasteiz, Spain; 3 Ikerbasque, Basque Foundation for Science, 48013 Bilbao, Spain

**Keywords:** kinematic viscosity, brines, sub-zero temperatures, CO2RR

## Abstract

The current race for space exploration has hastened the development of electrochemical technologies for the in-situ utilisation of planetary resources for the synthesis of vital chemicals such as O_2_ and fuels. Understanding the physicochemical properties, such as the density and kinematic viscosity, of aqueous solutions is essential for the design of electrochemical devices for the electrolysis of water and CO_2_, particularly at low temperatures. The density and kinematic viscosity of highly concentrated Mg(ClO_4_)_2_ and KOH solutions have been determined, both at low temperatures and in the presence of CO_2_ gas. It was found that, for all of the solutions, independent of the concentration or nature of the electrolyte, as the temperature was decreased to 255 K, the density and the viscosity of the solutions increased. Upon saturation with CO_2_, no significant change to the density and viscosity of Mg(ClO_4_)_2_, at all of the temperatures measured, was observed. Conversely, the CO_2_ saturated solutions of KOH showed significant changes in density and viscosity at all temperatures, likely due to the formation of carbonates. The effects of these changes on the diffusion coefficient for dissolved CO_2_ is also discussed.

## 1. Introduction

The current exploration of extra-terrestrial surfaces relies on unmanned probes, such as Curiosity on the surface of Mars, Rosetta and its associated lander, Philae, which landed on the comet Churyumov–Gerasimenko, and the Voyager probes. However, human exploration and colonisation to the farthest reaches of our solar system face many challenges, which need to be overcome. To explore and colonise other planets for extended periods of time, human astronauts will need essential supplies, such as oxygen and chemicals that can be used as fuels. However, there are prohibitive weight limits to space travel; therefore, the practice of in-situ resource utilisation to generate products with local materials is essential in this endeavour. For example, future plans for the exploration of the Martian surface by humans involve refueling the ascent vehicles on the surface using in-situ resources. Transporting the necessary fuel from Earth would require almost 40 Mt of oxygen and methane, which clearly poses a significant challenge in weight transport [1,2] However, the first experiments to generate oxygen on the surface have already taken place. By December 2021, the Mars Oxygen In Situ Resource Utilisation experiment (MOXIE) had generated around 50 g of O_2_ via a solid oxide fuel cell at 800 °C from the compressed Martian atmosphere [1].

Other electrocatalytic processes could be carried out to produce the necessary chemicals, such as hydrogen or methane, through the electrolysis of CO_2_ dissolved in water [3,4]. The Phoenix lander observed gulley formations on the surface of Mars, likely caused by the flow of liquid over the surface, which, coupled with soil analysis, led to the conclusion that perchlorate brines exist under certain conditions on the surface of Mars [5,6,7,8,9,10,11]. Previous research has shown that lowering the temperature below 0 °C increases the production of CH_4_ and CO and decreases the production of H_2_ when CO_2_ is electrocatalytically reduced in brines of Mg(ClO_4_)_2_ [3]. The non-standard temperature and pressure conditions of extra-terrestrial regions need to be considered when designing catalytic processes, and they can sometimes be advantageous. 

Parameters such as the pressure, temperature and electrolyte salinity are a rich area of research for the electrocatalytic conversion of CO_2_ on Earth [12,13,14] The ever-increasing concentration of CO_2_ in our atmosphere is causing climate change at an alarming rate. One avenue to mitigate the excessive accumulation of CO_2_ in the atmosphere is to capture CO_2_ at major production sites and either store it underground as CO_2_ clathrates or convert it to value-added products [15,16,17,18,19,20]. The electrocatalytic reduction of CO_2_ is one of many potential methods of CO_2_ conversion. Using concentrated electrolytes and low temperatures for a CO_2_ reduction reaction (CO2RR) is another avenue to increase CO_2_ solubility. Up to a point, the addition of salts to water lowers the freezing point, which means that electrocatalytic reactions can be carried out in the liquid phase at sub-zero temperatures [21,22,23,24,25]. 

It has been demonstrated that electrochemical processes can be carried out in solid aqueous electrolytes [26,27]; however, by increasing the electrolyte concentration, the freezing point of the solution can be depressed to maintain a liquid phase, which is more technologically advantageous. Blagden’s Law, Equation (1), can be used to estimate the depression in the freezing point of the electrolyte, Δ*T*, where *K* is the cryoscopic constant of the solvent, *m* is the molality (moles solute per kg solvent) and *i* is the Van’t Hoff factor, which describes the number of ions a species forms when fully dissociated.

(1) ∆T=K×m×i

Care should be taken when using Equation (1) as it does not account for the chemical activity of the ions and is only applicable for ideal solutions. Equation (1) can be used for general approximations, such as for the freezing point of sea water; however, at high concentrations, non-linear behaviour occurs and the freezing point begins to rise again [28,29]. Using brines as electrolytes enables the depression of the freezing point whilst, in theory, also favouring the increase in the solubility of certain gases, such as CO_2_, methane and O_2_ [30]. As such, the increase in the reactants might also increase the rate of the electrochemical reaction involving these gases. For example, in Mg(ClO_4_)_2_ brines, as the temperature is lowered to −35 °C, the solubility of O_2_ increases to around 1.1 mM and the current, due to O_2_ reduction, increases [31]. In previous works, we have also reported an increase in the reaction rate of the electrochemical conversion of CO_2_ and methane at sub-zero temperatures in aqueous brines of Mg(ClO_4_)_2_ and KOH [3].

At high pressures or low temperatures, gas clathrates are formed, where cages of water molecules encapsulate gas molecules [32]. These phases are well-known as, in the mining industry, they can form in gas pipelines, causing costly blockages, and because CH_4_ versions are found in permafrost or in the deep ocean. The CH_4_ hydrates found in the ocean and permafrost are a double-edged sword; they could be a huge reservoir of energy if electrocatalytic technology can be developed to exploit them, but there is a small possibility that if oceanic temperatures continue to rise, the hydrates may become unstable, releasing CH_4_ into the atmosphere and leading to runaway warming [33,34]. Several groups have explored the opportunity recovery of energy from CH_4_ clathrates and simultaneously sequestrated CO_2_ as clathrates through the direct swapping of CH_4_ by CO_2_ in one clathrate cavity. 

Changes in temperature not only have an effect on the solubility of CO_2_ in aqueous electrolytes, but they also have a marked effect on the dynamic viscosity, *η*, and the density, *ρ*. These factors are not only important in the engineering of future technology, but also for fundamental processes such as the mass transport of ions. The Nernst-Plank Equation (2) is used to describe the mass transfer to an electrode, encompassing the processes of diffusion, convection and migration.

(2) $$J_{i}\left(x\right)=-D_{i}\frac{\partial C_{i}\left(x\right)}{\partial x}-\frac{z_{i}F}{RT}D_{i}C_{i}\frac{\partial \phi \left(x\right)}{\partial x}+C_{i}v\left(x\right)$$

where at a distance *x* from an electrode for a species *i*, *J* is the flux (mol s^−1^ cm^−2^), *D* is the diffusion coefficient (cm^2^ s^−1^), *C* is the concentration (mol cm^−3^), *z* is the charge, *F* is the Faraday constant, *R* is the gas constant, *T* is the temperature (K), *φ* is the electrostatic potential (V) and *v* is the velocity (cm s^−1^). The diffusion coefficient, *D*, can be derived from the Einstein-Stokes Equation (3),

(3) $$D=\frac{k_{B}T}{4\pi \eta r_{eff}}$$

where *k_B_* is the Boltzmann constant, *r_eff_* is the effective radius of the species (m) and *η* is the dynamic viscosity of the solution (Pa s). The dynamic viscosity is derived from the kinematic viscosity, *ν* (m^2^ s^−1^), and the density via Equation (4).

(4) $$\eta \;\left(\mathrm{Pa}\;s\right)=\nu \;\left(m^{2}{\;s}^{-1}\right)\times \rho \;\left({\mathrm{kg}\;m}^{-3}\right)$$

when designing electrolysers for any catalytic process involving a liquid reaction media, the viscosity of the media is important for the physical design aspects as the viscosity will affect the flow-through components. However, as Equation (2) shows, the flux of any reactants or products of the reaction is heavily influenced by the diffusion coefficient, which is, in-turn, heavily affected by the viscosity. Given the importance of the viscosity for mass transport during electrochemical reactions, such as CO_2_ conversion [35,36,37,38] and water splitting [39], herein we report the values of the density and dynamic viscosity at sub-zero temperatures down to 255 K for Mg(ClO_4_)_2_ and KOH brines in the absence of, and upon saturation with, CO_2_ gas.

These results can then be used to better understand the anti-Arrhenius behaviour seen during the electrochemical conversion of CO_2_ and the oxidation of methane at temperatures below −5 °C reported in our previous work [3]. A more complete understanding of the electrochemical conversion of CO_2_ at low temperatures will further promote the design of electrochemical devices for space exploration and colonisation.

## 2. Results and Discussion

### 2.1. Determination of the Density of Mg(ClO_4_)_2_ and KOH Solutions as a Function of Temperature

**Figure 1:**
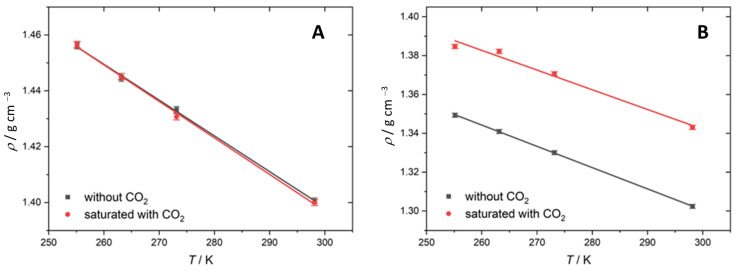
Density as a function of temperature between 255 K and 298 K with CO_2_ (red) and without CO_2_ (black). (**A**) Results for 3.8 m Mg(ClO_4_)_2_. (**B**) Results for 8.5 m KOH.

Figure 1 shows the densities of the Mg(ClO_4_)_2_ and KOH solutions as a function of the temperature. As expected, the density of all the solutions increases linearly with the decreasing temperature. At 255 K, 3.8 mol kg^−1^ is close to the saturation of the Mg(ClO_4_)_2_ solution, and below this temperature, precipitation of 6H_2_O·Mg(ClO_4_)_2_ occurs [28], which is the most stable form under Martian conditions [40]. Upon CO_2_ saturation, the solution of 3.8 m Mg(ClO_4_)_2_ showed no significant change in the density at any T measured, suggesting little absorption of CO_2_ in the solution. These results are in agreement with the poor solubility of CO_2_ at the pH of the Mg(ClO_4_)_2_ solutions (pH = 8). The previous work by our group using mass spectroscopy found that, in the same electrolyte, the concentration of CO_2_ increased from 1.12 mM at 293.15 K to 5.08 mM at 253 K [3]. However, such small changes in the concentration of CO_2_ in the solution are below the detection limit and within the standard deviation of the measurements using the methodology used in this work.

Upon saturation with CO_2_, the density of 8.5 m KOH increases by 0.04 g cm^−3^ across all the measured temperatures (Table 1). This indicates that the amount of CO_2_ absorbed is constant over all temperatures in this range. Alternatively, as in the case of the Mg(ClO_4_)_2_, the variation in the concentration of CO_2_ is, again, too small to be seen via this methodology. In this regard, previous works have shown that, at room temperature, the concentration of absorbed CO_2_ increases with the concentration of KOH [41] due to the formation of (bi)carbonates, but at very high concentrations of the electrolyte, the CO_2_ solubility is limited due to the so-called “salting out” effect [42,43]. The balance between these factors—that is, (bi) carbonate formation and the salting out effect—and the expected increase in the CO_2_ solubility due to the lower temperature may account for the observed constant CO_2_ concentration across the temperatures measured.

### 2.2. Determination of the Viscosity of the Mg(ClO_4_)_2_ and KOH Solutions as a Function sof Temperature

**Figure 2:**
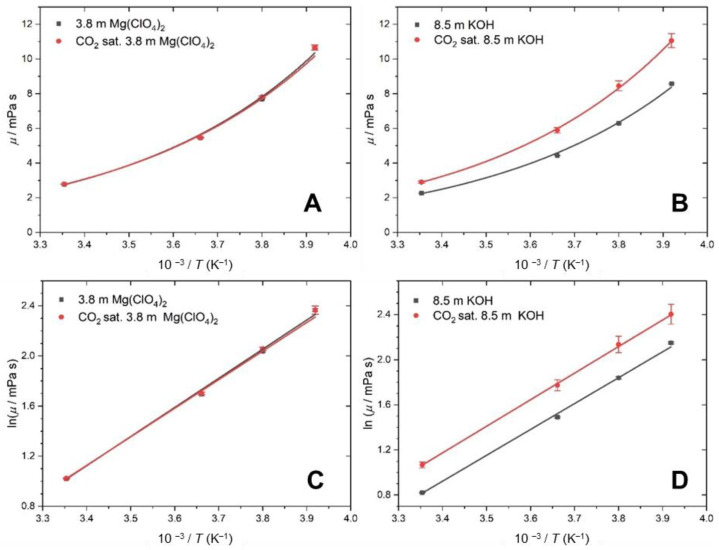
Viscosity as a function of temperature (black) without CO_2_ and (red) upon saturation with CO_2_. (**A**,**C**) Results for 3.8 m Mg(ClO_4_)_2_. (**B**,**D**) Results for 8.5 m KOH.

The measured kinematic viscosity, *ν*, was converted to the dynamic viscosity, *η*, via Equation (4), and the results are shown in Table 2. For *ν*, the expanded uncertainty was 6 × 10^−4^ mm s^−1^. Over the temperature range investigated, the values of the viscosity for both the Mg(ClO_4_)_2_ and KOH solutions fit an Arrhenius relationship, Equation (5),

(5) $$\eta =Ae^{\frac{E_{a}}{RT}}$$

where *R* is the gas constant, *A* is the preexponential factor (Pa s) and *E_a_* is the activation energy (kJ mol^−1^). The Arrhenius plots are shown in Figure 2A,B. Figure 2A shows little difference between *η* for the CO_2_ saturated and unsaturated Mg(ClO_4_)_2_ solution. This agrees with the interpretation of the *ρ* results, where there is little absorption of CO_2_. Previous work has attributed the increase in viscosity for concentrated Mg(ClO_4_)_2_ solutions to the formation of solvation spheres on a picosecond timescale around the ions, which act as suspended spheres in the solution, which in turn increases the viscosity [44].

On the other hand, Figure 2B shows an increase in the difference in *η* for the unsaturated and CO_2_ saturated KOH solutions as the temperature decreases. As *ρ* indicates that the concentration of CO_2_ is constant across the temperature range, the change in *η* must be due to changes in the strength of the intermolecular forces of attraction. When the CO_2_ dissolves, it reacts with the available water and sets up an equilibrium between CO_3_^2−^, HCO_3_^−^ and H_2_CO_3_. In concentrated KOH, the most abundant ion is HCO_3_^−^, which causes an associated lowering of the pH [41]. The carbonate ions can form hydrogen bonds and interact with the wider water network, which increases the intermolecular forces of attraction, and therefore the viscosity.

In order to obtain the activation energy of the system, Figure 2C,D show the Arrhenius linear representation of the measured values of the viscosity as a function of the temperature using Equation (6).

(6) $$\ln\eta =\ln\eta_{\infty }+\frac{E_{a}}{R}\cdot \frac{1}{T}$$

*η_∞_* can be interpreted as the viscosity of the solution at infinite temperature (Pa s) and *E_a_* is the energy input needed for molecules in the solution to flow past each other. These parameters are derived for each of the solutions, as shown in Table 3. There is a small increase in *E_a_* when KOH is saturated with CO_2_, which supports the idea that there is an increase in the strength of the interactions between the ions.

### 2.3. Determination of the Diffusion Coefficient of CO_2_ in Mg(ClO_4_)_2_ and KOH

Using the values for *η* determined in the previous section and Equation (3), the values for *D* were calculated, and the results are shown in Table 4. The hydrodynamic radius of the CO_2_ was assumed to be the same as in pure water [45], but loosely dependent on the temperature [46] As T decreases, the diffusion coefficient also decreases in both solutions. The larger *η* values for 8.5 m KOH correspond with lower *D* values compared to 3.8 m Mg(ClO_4_)_2_. According to Equation (2), the lower values of the diffusion coefficient indicate a lower flux and a decrease in the mass transport of CO_2_ through the solutions. The results obtained using pulsed-field gradient 13C NMR showed a trend of a decrease in *D* with increasing salinity of the brines; for example, 2.06 10^−9^ m^2^ s^−1^ in 1.0 m NaCl decreasing to 1.29 10^−9^ m^2^ s^−1^ in 5.0 m NaCl [45]. The results obtained using a Taylor dispersion method show that *D* also decreases with the temperature in pure water [46]; therefore, the results presented in Table 4 fit with the trends presented in the literature.

Interestingly, our previous work showed an increase in the electrochemical conversion of CO_2_ in brines at sub-zero temperatures, with and without mass transport control. Therefore, even though the physicochemical properties of the solution, such as the density and viscosity, influence the diffusion of the gaseous reactant species, other intrinsic parameters promote Anti-Arrhenius behaviour so that, at low temperatures, the catalytic activity increases. 

## 3. Materials and Methods

### 3.1. Preparation of Materials

Solutions of KOH (Sigma Aldrich, Gillingham, UK > 85%) and Mg(ClO_4_)_2_ (Alfa Aesar, Heysham, UK > 95%) were prepared by weighing out the required mass of solid using an analytical balance (measuring to 0.01 mg) and dissolving in a weighed mass of ultrapure water (Millipore Milli-Q^®^ Integral 3, 18.2 MΩ cm^−1^, <5 ppb total organic carbon) to make the correct concentration in mol kg^−1^. All solutions were left in a temperature controlled 1:1 glycerol:water bath and cooled using a HUBER TC45E cooler with a temperature control ±0.5 K until they reached the desired temperature. Solutions were saturated with CO_2_ (BOC, N4 grade) by bubbling for 20 min at room temperature and pressures of 0.5 bar, and then for another 10 min at each temperature before each measurement was taken. Given the units involved in Equation (1), all the concentrations in the manuscript are reported in molality (moles solute per kg solvent). The supplier and purity of the reactants are included in Table 5.

### 3.2. Density Measurements

Pycnometers (±0.0100 cm^3^) were acquired from Fisher Scientific and calibrated using acetonitrile (Fisher Scientific, Morecambe, UK, 99.9%, Extra Dry over Molecular Sieves) at 298 K, 273 K, 263 K and 255 K. For density measurements, the pycnometers were filled with solutions that had been pre-cooled in the temperature-controlled bath and then left again to reach the desired temperature before measurements were taken (usually > 10 min). The reported densities for 8.5 m KOH and 3.8 m Mg(ClO_4_)_2_ are an average of at least 12 measurements from three different samples. The reported error is calculated via the root sum of the squares of the error in the pycnometer and the error in the calibration curve fit (error in volume of pycnometer ± 3 × 10^−4^ cm^−3^).

### 3.3. Viscosity Measurements

To measure the kinematic viscosity, an Ubbelohde viscometer (Paragon Scientific, Birkenhead, UK, ±0.17%) suspended in the same temperature-controlled bath was used. When solutions were introduced to the viscometer, the sample was again left to cool to temperature before any measurement took place. The flow time was measured with a digital stopwatch capable of measuring ±0.1 s. Kinematic viscosity (*ν*) was calculated using the equation,

(7) $$\upsilon \;\left({\mathrm{mm}}^{2}s^{-1}\right)=A\;\left({\mathrm{mm}}^{2}{\;s}^{-2}\right)\times t\;\left(s\right)$$

where *t* is the flow time and *A* is the calibration constant of the viscometer. Kinematic viscosities are an average of at least 15 measurements from three different samples and the standard deviation of the measurements taken as the error.

The measured kinematic viscosity, *ν*, was converted to the dynamic viscosity, *η*, via Equation (4) and the errors propagated via root sum of the squares.

### 3.4. Methodology Validation

To validate the methodology, density and viscosity measurements were determined for pure water and KOH solutions and are compared to previous works. As can be seen in Table 6, in the case of water, both the density and the dynamic viscosity are within 1% of the literature results [47]. For 8.5 m KOH, the density value reported in this work is within 1% of the literature value; however, the dynamic viscosity has a 4% deviation [48]. Such an error can be associated with the hygroscopic nature of KOH solutions causing a decrease in the concentration of the solutions during measurement of the viscosity. 

For 3.8 m Mg(ClO_4_)_2_, there are few values in the literature with which to compare. However, for 4.23 m Mg(ClO_4_)_2_ at 298 K, Sohnel et al. recorded a value of *ρ* = 1.4373 g cm^−3^ [49]. Herein, we record *ρ* = 1.4006 g cm^−3^ at the same temperature for the lower concentration of 3.8 m. Similarly, for 8.21 m KOH at 273 K, Kelly et al. recorded *ρ* = 1.4373 g cm^−3^ and *η* = 4.409 mPa s [50]. In comparison, the values recorded herein for 8.5 m KOH at 273 K are 1.3300 g cm^−3^ and 4.44 mPa s. Again, we ascribe deviations to the hygroscopic nature of concentrated KOH solutions.

## 4. Conclusions

The density and dynamic viscosity of the highly concentrated KOH and Mg(ClO_4_)_2_ solutions were measured and reported at temperatures below 273 K. The high electrolyte concentration results in the depression of the freezing point of the aqueous solution down to 255 K. It was found that as the temperature decreased, the *η* and *ρ* for all the solutions increased. The *ρ* increased linearly and the *η* followed an Arrhenius relationship. Given the importance of these solutions for the development of electrolysers on other planetary objects, the density and dynamic viscosity of the same KOH and Mg(ClO_4_)_2_ solutions saturated with CO_2_ are also reported. 

The 3.8 m Mg(ClO_4_)_2_ showed no significant differences in *η* or *ρ* at any measured temperature after saturation with CO_2_. The results are associated with the poor solubility of CO_2_ at the pH of the Mg(ClO_4_)_2_. Conversely, the 8.5 m KOH solutions showed marked changes in their physicochemical behaviour upon saturation with CO_2_. A constant increase in the *ρ* was observed; at the same time, a much greater increase was observed in the *η* at lower temperatures. We conclude that the formation of (bi) carbonate species due to the reaction between the CO_2_ and KOH increased the strength of the interactions between the ions, resulting in a larger change in the viscosity at lower temperatures. 

At low temperatures, the diffusion coefficient of CO_2_ is significantly reduced in these solutions, which will result in less mass transport through the electrolyte. This indicates that the increase in the CO_2_ reduction current at low temperatures seen in our previous work is due to an increase in the kinetics of the reaction, not an increase in the transport of CO_2_ to the electrode surface [3]. However, the benefit of the increased activity at low temperatures is finely balanced with the disadvantageous increase in the viscosity of the solutions and the decrease in the diffusion of CO_2_. 

On the basis of these results, we believe that the increase in the electrochemical activity in the electrochemical reduction of CO_2_, and possibly the oxidation of CH_4_ at low temperatures [3], could be related to the large DC electric field near the surface due to changes in the double layer (DL) associated with the high concentration of the electrolyte and the low temperature. The electric field from the solvated cations in the DL can substantially favour the formation of key intermediates of the reactions, thus increasing the kinetics of the reaction [51]. These insights are crucial for the further investigation of catalytic reactions at low temperatures in brines.

## Figures and Tables

**Table 1:** Measured density of 3.8 m Mg(ClO_4_)_2_ and 8.5 m KOH solutions, with and without CO_2_ saturation.

	3.8 m Mg(ClO_4_)_2_	CO_2_ sat. 3.8 m Mg(ClO_4_)_2_	8.5 m KOH	CO_2_ sat. 8.5 m KOH
T/K	*ρ*/g cm^−3^			
298	1.4006 ± 0.0011	1.4000 ± 0.0011	1.3024 ± 0.0010	1.3430 ± 0.0011
273	1.4332 ± 0.0011	1.4307 ± 0.0011	1.3300 ± 0.0010	1.3706 ± 0.0011
263	1.4445 ± 0.0011	1.4451 ± 0.0011	1.3409 ± 0.0011	1.3821 ± 0.0011
255	1.4562 ± 0.0012	1.4566 ± 0.0012	1.3493 ± 0.0011	1.3847 ± 0.0011

Standard uncertainty for T = 0.5 K. Standard uncertainty for *ρ* is in the table.

**Table 2:** Calculated *η* values for Mg(ClO_4_)_2_ and KOH solutions with and without saturation with CO_2_.

	3.8 m Mg(ClO_4_)_2_	CO_2_ sat. 3.8 m Mg(ClO_4_)_2_	8.5 m KOH	CO_2_ sat. 8.5 m KOH
T/K	*η*/mPa s			
298	2.77 ± 0.04	2.78 ± 0.04	2.27 ± 0.03	2.91 ± 0.08
273	5.45 ± 0.09	5.48 ± 0.07	4.44 ± 0.06	5.89 ± 0.18
263	7.67 ± 0.10	7.82 ± 0.11	6.29 ± 0.09	8.45 ± 0.31
255	10.65 ± 0.14	10.66 ± 0.2	8.58 ± 0.11	11.06 ± 0.43

Standard uncertainty for T = 0.5 K. Standard uncertainty for *η* are in the table.

**Table 3:** Viscosity at infinite temperature and the activation energy for each solution derived from the linear fit in Figure 2C,D.

Solution	10^−3^ *η_∞_*/mPa s	*E_a_*/kJ mol^−1^	R^2^ Values for Figure 2C,D
3.8 m Mg(ClO_4_)_2_	1.14 ± 0.03	19.3 ± 0.4	0.999
CO_2_ sat. 3.8 m Mg(ClO_4_)_2_	1.32 ± 0.04	19.0 ± 0.4	0.998
8.5 m KOH	1.03 ± 0.03	19.1 ± 0.5	0.998
CO_2_ sat. 8.5 m KOH	1.07 ± 0.02	19.6 ± 0.3	0.999

**Table 4:** Calculated diffusion coefficient for CO_2_ in 3.8 m Mg(ClO_4_)_2_ and 8.5 m KOH at various temperatures.

	CO_2_ sat. 3.8 m Mg(ClO_4_)_2_	CO_2_ sat. 8.5 m KOH
T/K	*D*/10^−10^ m^2^ s^−1^	
298	7.01 ± 0.03	6.71 ± 0.2
273	3.43 ± 0.02	3.19 ± 0.09
263	2.37 ± 0.02	2.19 ± 0.07
255	1.71 ± 0.02	1.65 ± 0.06

Standard uncertainties for D reported in table. Standard uncertainty for T = 0.5 K. Expanded uncertainty for D in 3.8 m Mg(ClO_4_)_2_ = 4 × 10^−14^ and in 8.5 m KOH = 2 × 10^−13^ (0.95 level of confidence).

**Table 5:** CAS registry number, supplier and purity of materials used in this work.

Component	CAS Reg. No.	Supplier	Purity
KOH	1310-58-3	Sigma Aldrich	>85%
Mg(ClO_4_)_2_	10034-81-8	Alfa Aesar	>95%
CH_3_CN	75-05-8	Fisher Scientific	99.9%

**Table 6:** Comparison between density and viscosity measurements obtained in this work and in previous reports. ^a^ Reference [47]. ^b^ Calculated from reference [48].

	*Ρ* at 298 K/g cm^−3^		*η* at 298 K/mPa s	
	This Work	Literature	This Work	Literature
water	1.0005 ± 0.0008	0.997 ^a^	0.900 ± 0.004	0.890 ^a^
8.5 m KOH	1.3024 ± 0.0010	1.309 ^b^	2.271 ± 0.008	2.362 ^b^

Standard uncertainty for T = 0.5 K. Standard uncertainty for *ρ* and *η* are given in the table. Expanded uncertainty for water, U(*ρ*) = 2 × 10^−5^ g cm^−3^, U(*η*) = 1 × 10^−4^ mPa s. Expanded uncertainty for 8.5 m KOH, U(*ρ*) = 3 × 10^−5^ g cm^−3^, U(*η*) = 1 × 10^−4^ mPa s. (0.95 level of confidence).

## Data Availability

Not applicable.

## Abbreviations

MOXIE	Mars Oxygen In Situ Resource Utilisation experiment
CO2RR	CO_2_ reduction reaction
Δ*T*	depression in freezing point of the electrolyte
*K*	cryoscopic constant of the solvent
*m*	molality (moles solute per kg solvent)
*i*	Van’t Hoff factor
*η*	dynamic viscosity (mPa s)
*ρ*	density (g cm^−3^)
*J*	flux (mol s^−1^ cm^−2^)
*D*	diffusion coefficient (cm^2^ s^−1^)
*C*	is the concentration (mol cm^−3^)
*z*	charge
*F*	Faraday constant
*R*	gas constant
*T*	temperature (K),
*φ*	electrostatic potential (V),
*v*	velocity (cm s^−1^)
*ν*	kinematic viscosity (mm^2^ s^−1^)
*A*	preexponential factor (Pa s),
*E_a_*	activation energy (kJ mol^−1^)
